# Opportunities to Enhance the Implementation of Veterans Affairs Video-Based Care: Qualitative Perspectives of Providers from Diverse Specialties

**DOI:** 10.2196/43314

**Published:** 2023-04-24

**Authors:** Cindie Slightam, Charlie Wray, Rebecca L Tisdale, Donna M Zulman, Caroline Gray

**Affiliations:** 1 Center for Innovation to Implementation Veterans Affairs Palo Alto Health Care System Menlo Park, CA United States; 2 Department of Medicine University of California San Francisco, CA United States; 3 Hospital Medicine San Francisco Veterans Affairs Medical Center San Francisco, CA United States; 4 Department of Health Policy Stanford University School of Medicine Stanford, CA United States; 5 Department of Primary Care and Population Health Stanford University School of Medicine Stanford, CA United States

**Keywords:** implementation, video visit, VA, qualitative, adoption, perspective, health care provider, physician, health care professional, veteran, virtual visit, virtual care

## Abstract

**Background:**

Increasing the adoption of digital care tools, including video visits, is a long-term goal for the US Department of Veterans Affairs (VA). While previous work has highlighted patient-specific barriers to the use of video visits, few have examined how clinicians view such barriers and how they have overcome them during the rapid uptake of web-based care.

**Objective:**

This study sought input from providers, given their role as critical participants in video visit implementation, to qualitatively describe successful strategies providers used to adapt their practices to a web-based care setting.

**Methods:**

We conducted interviews with 28 VA providers (physicians and nurse practitioners) from 4 specialties that represent diverse clinical services: primary care (n=11), cardiology (n=7), palliative care (n=5), and spinal cord injury (n=5). All interviews were audio recorded and transcribed, and transcripts were reviewed and coded according to an iteratively created codebook. To identify themes, codes were grouped together into categories, and participant comments were reviewed for repetition and emphasis on specific points. Finally, themes were mapped to Expert Recommendations for Implementing Change (ERIC) strategies to identify evidence-based opportunities to support video visit uptake in the VA.

**Results:**

Interviewees were mostly female (57%, 16/28), with an average age of 49 years and with 2-20 years of experience working in the VA across 16 unique VA facilities. Most providers (82%, 23/28) worked in urban facilities. Many interviewees (78%, 22/28) had some experience with video visits prior to the COVID-19 pandemic, though a majority (61%, 17/28) had conducted fewer than 50 video visits in the quarter prior to recruitment. We identified four primary themes related to how providers adapt their practices to a web-based care setting: (1) peer-based learning and support improved providers’ perceived value of and confidence in video visits, (2) providers developed new and refined existing communication and clinical skills to optimize video visits, (3) providers saw opportunities to revisit and refine team roles to optimize the value of video visits for their care teams, and (4) implementing and sustaining web-based care requires institutional and organizational support. We identified several ERIC implementation strategies to support the use of video visits across the individual-, clinic-, and system-levels that correspond to these themes: (1) individual-level strategies include the development of educational materials and conducting education meetings, (2) clinic-level strategies include identifying champions and revising workflows and professional roles, and (3) system-level strategies include altering incentive structures, preparing implementation blueprints, developing and implementing tools for quality monitoring, and involving executive leadership to encourage adoption.

**Conclusions:**

This work highlights strategies to support video visits that align with established ERIC implementation constructs, which can be used by health care systems to improve video visit implementation.

## Introduction

The Department of Veterans Affairs (VA) health care system comprises 170 medical centers and more than 1000 community-based outpatient clinics across the United States. The VA manages over 9 million enrolled veterans, 30% of whom reside in rural regions [1]. The VA is a leader in video visit implementation and use in the United States and provided 3.8 million video visits to veterans in the fiscal year 2020 [2]. The COVID-19 pandemic dramatically increased the need for video visits across the VA and among other health care systems across the United States [3-6]. At the start of the pandemic, video visits across primary and specialty care services increased more than 10-fold in the VA [7,8].

While a variety of benefits to video visits have been reported for both patients (increased access [9], reduced travel, and lower cost [10]) and providers (seeing the home environment [11,12]), video visit adoption continues to vary across the network of VA medical centers and specialties since the initial uptake early in the pandemic [13,14].

In an effort to enhance and build on the structures and resources developed during the pandemic, a long-term goal of the VA is to continue to improve on the adoption of video visits. Despite this focus, common barriers still exist when providing video-based care [15,16], highlighting opportunities to address barriers for patients (eg, lack of access to technology, digital skill needs, and privacy concerns), providers (eg, knowledge, training, and concerns about efficacy), and clinics (eg, scheduling difficulties and workflow inefficiencies) [12,16-22]. Additional barriers at the system and organizational level include patient engagement, operational workflow, organizational readiness, and regulatory changes to sustain outpatient telehealth programs [23].

Several projects have examined provider perspectives on sustaining the growth of telemedicine [12,24,25]. Training and education are clearly important, and there are a growing number of program guides to support the implementation of telehealth [26,27]. However, while recent work has underscored the system and organizational issues that may impact the use of video visits, there remains a need to understand how providers, despite these challenges, implement video visits successfully. These experiences can then be translated into strategies that support video visit implementation and sustained use [12,25,28].

Given that providers have a vested interest in the success of video visits, we sought to identify the strategies that VA providers endorse to effectively provide care via video visits, as well as opportunities for improving the use of video visits. Using qualitative methods, we describe strategies that providers used to successfully provide video care and opportunities to integrate them post pandemic.

## Methods

### Overview

Our team conducted qualitative interviews with providers from across the VA to describe their experiences delivering video-based care. We used a combination of administrative data and provider referrals to purposefully sample 28 providers [29], ranging in specialties from primary care (n=11), cardiology (n=7), palliative care (n=5), and spinal cord injury (n=5). These specialties were identified to represent a diverse set of clinical care and services that can be offered during a video visit [30]. Primary care addresses a patient’s medical, social, and behavioral needs through history-taking, physical examinations, laboratories, imaging, and medication management [31]; cardiology care focuses on a patient’s cardiac conditions and offers specialized procedures [32]; palliative care typically centers around in-depth goals of care discussions and medication management near the end of life [33]; and spinal cord injury care provides comprehensive education, counseling, coordination, and ongoing monitoring for a highly complex chronic condition [34]. Providers were identified from administrative data based on being in the top quartile of the number of video visits provided in the 3-6 months (for their specialty by facility) prior to recruitment. Providers were sampled from several US regions and a mix of urban and rural facilities.

To learn about their experiences offering video visits prior to and during the COVID-19 pandemic, 2 researchers (CG and RT) conducted interviews with providers. Interviews took place between December 2020 and June 2021 over Microsoft Teams (version 1; Microsoft Corporation). Based on a review of existing literature as well as engagement with prior web-based care projects conducted at the VA, the research team identified provider perspectives as a noticeable gap. A semistructured interview guide was created, featuring exploratory questions meant to elicit providers’ overall experiences with video care, challenges, facilitators, and recommendations for optimizing web-based care delivery (see Multimedia Appendix 1 for the interview guide). Interview questions focused on the provider’s choice to offer video visits, preferences for providing care digitally or in person, skills and training required, and perceived barriers to providing care via video visits. Participants were asked to provide verbal consent prior to interviews. Interviews lasted approximately 30 minutes and were recorded and transcribed by a professional transcription service.

We used a descriptive-qualitative approach involving constant comparison [35,36] to synthesize the interview data. The research team first reviewed 5 transcripts, identified emergent codes, and created a codebook to code all transcripts. All transcripts were coded in Atlas.ti (version 9.1; ATLAS.ti Scientific Software Development GmbH) according to the codebook. Codes and associated text were grouped into categories, after which all text was reviewed for recurrence and emphasis on specific points. All team members participated in selecting exemplary quotes and sorting themes into categories.

Lastly, to further support the implementation and uptake of video visits, challenges and recommendations identified in the interviews and coded to themes were also mapped to the Expert Recommendations for Implementing Change (ERIC) framework, an implementation science framework that summarizes evidence-based implementation strategies that have been demonstrated to enhance the adoption, implementation, scale-up (or spread), and sustainment of a program or practice [37,38]. The ERIC framework includes 73 discrete implementation strategies across 9 functional categories. The challenges and recommendations were categorized to identify the appropriate intervention level necessary to address the issue based on their impact at the provider (individual), clinic, and VA system levels.

### Ethical Considerations

This work was conducted as part of a VA–funded evaluation that was designated as nonresearch by the VA’s Office of Rural Health and was exempt from institutional review board and research and development committee review.

## Results

### Overview

Twenty-six physicians and 2 nurse practitioners agreed to participate. Table 1 describes the characteristics of the providers. The majority were female (n=16), with a mean age of 49 (SD 10) years. All interviewees had worked at the VA for at least 2 years (ranging from 2 to 20 years). The providers represented 16 VA facilities across the United States, and many worked in high-complexity (n=11) and urban (n=23) facilities. Their experience with video visits ranged from several years to recent adoption; a minority stated that the COVID-19 pandemic introduced them to video visits (n=6). Less than half of the providers had conducted more than 50 video visits in the quarter prior to recruitment (n=11).

**Table 1:** Characteristics of health care providers (N=27)^a^.

Characteristics		Values
Age (years)^b^, mean (SD), n=24		49 (10)
Female, n (%)		16 (59)
**Facility setting^b^, n (%)**		
	Rural	5 (18)
	Urban or suburban	23 (82)
**Facility complexity^b,c^, n (%)**		
	Highest (1a, 1b, 1c)	22 (81)
	Medium	3 (11)
	Low (3)	2 (7)
**Clinical specialty^b^, n (%)**		
	Primary care	11 (39)
	Cardiology	7 (25)
	Spinal cord injury	5 (18)
	Palliative care	5 (18)
**Clinic visits per quarter^b,d^, n (%)**		
	0-50	13 (48)
	51-100	5 (19)
	100+	9 (33)
**Video visits per quarter^b,d^, n (%)**		
	0-50	16 (60)
	51-100	11 (40)

^a^Missing data from 1 participant.

^b^Identified from VA Corporate Data Warehouse [39].

^c^Facility Complexity Model is based on patient population, clinical service complexity, and education and research funding [40].

^d^Visits between April 1 and June 30, 2020.

Providers described numerous strategies that they used or felt would be helpful to overcome barriers when implementing video visits. These are described in four themes: (1) peer-based learning and support improved providers’ perceived value of and confidence in video visits, (2) providers developed new and refined existing communication and clinical skills to optimize video visits, (3) providers saw opportunities to revisit and refine team roles to optimize the value of video visits for their care teams, and (4) implementing web-based care initiatives requires institutional and organizational support. In addition to collating overarching themes, we mapped individual-, clinic-, and system-level challenges and recommendations to the ERIC implementation framework in order to identify implementation strategies for further testing and research.

### Theme 1: Peer-Based Learning and Support Improved Providers’ Perceived Value of and Confidence in Video Visits

Providers described how early in the pandemic, they and their colleagues sought guidance and training on how to provide effective video-based care. Due to a lack of evidence or guidance around video visit implementation and use, providers often relied on peer-to-peer knowledge to overcome initial barriers or challenges. A primary care provider details this perspective and highlights examples of specific peer-to-peer learning strategies used at their facility:

We do …. interactive sessions where we go through cases together and do breakout sessions so that the trainees can also practice with each other, simulating both being the patient and provider, trying to get through each—like the majority of [body] systems in terms of doing a physical exam primarily.

P1

Providers noted that the sharing of best practices encouraged providers who were initially ambivalent about offering video visits to try offering more video care. A palliative care provider described how daily huddles helped support and facilitate video visits:

A lot of the providers who are not used to telehealth, the telehealth team had offered what they call a noon huddle. So that brought all the providers who are really interested in learning more about how to manage, how to navigate, and you would literally get a day-to-day tutorial and new development of what is being provided through the tele or virtual.

P26

Huddles and other similar peer-based learning strategies demonstrate how peer support played a role in facilitating the adoption of video-based care among providers. Through these interactions, providers described how they were able to troubleshoot challenging clinical scenarios and patient needs, which increased their comfort with video-based care.

### Theme 2: Providers Developed New and Refined Existing Communication and Clinical Skills to Optimize Video Visits

There was broad agreement among providers that optimizing the provider-patient interaction over video required learning the technical aspects of the video visit and adapting communication and physical examination skills. Providers acknowledged that due to the technical challenges that video visits presented, it was important to allow for additional time and patience with patients. They also noted that adjusting their communication strategies to account for the video format facilitated successful visits, as this palliative care provider illustrates:

So, when I’m talking about [clinical concerns] virtually, I am frequently pausing, checking in, referencing the patient by their first name to make sure that they're involved, kind of slowly checking in for caregivers to participate in the conversation. So, a lot of this requires a lot more intentionality; whereas, in person I might just use eye contact just with that person or I’d touch them on the shoulder and see how they're feeling. There are a lot of different ways to check in on emotion when using nonverbal communication.

P28

Providers also described how they adapted their history-taking skills to ensure that necessary patient information was collected. For example, a cardiologist shared how video-based visits prompted them to approach patient interviews in a slightly different way than they would for a face-to-face visit, noting:

[Video visits have] prompted me to revisit just basic history-taking skills as well. You have to get a little bit creative about how you ask questions when you're trying to get to the bottom of whether this patient is really [experiencing a heart failure exacerbation].

P21

### Theme 3: Providers Saw Opportunities to Revisit and Refine Team Roles to Optimize the Value of Video Visits for Their Care Teams

Providers described the important roles all members of their clinical team play in improving video visit success and highlighted opportunities for other staff members to be involved. They noted how facilitating a sense of shared responsibility for video visits helped encourage more successful video visits. For example, 1 primary care provider explained:

I think the more we can involve each member of the care team and that everybody feels like they have a responsibility in ensuring … video literacy among patients, the more success we'll have over the long-term, because it pays it forward for everybody else.

P5

This provider noted the importance of ensuring video literacy among patients, which other providers similarly characterized as critical for successful implementation of video visits. Having other members of the clinical team assist patients with technology, particularly first-time users, was described as a major facilitator of video-based care success and uptake. Doing so helped alleviate the burden on the clinical team. Elaborating on this point, 1 provider explained how dedicated telehealth technicians helped patients become comfortable with video visits:

When we're talking about doing a video visit with a patient for the first time, having somebody here that can show them, “This is how you do it” and do a practice call with them before they leave the building. We have our telehealth technicians do practice calls with them or attempt to reach out if I've scheduled a [video visit].

P18

Providers advocated for the development of standard operating procedures that provide patients technical support while minimizing disruptions to visits.

Though acquiring new technical skills was necessary to succeed at video visits, providers nevertheless maintained that patients’ technical challenges often become a burden they must overcome. They acknowledged that troubleshooting during a video visit is necessary but felt that providers should not assume a technical support role. They suggested that reimagining existing roles or adopting new roles would better support patients’ technical needs, as a cardiologist explains below:

And what we're really trying to do is create a sharp divide between a provider being a health care provider and them being a technician. I think that the more that we can carve out technical stuff for people who wear that designated hat, I think that would be better…

P22

Furthermore, providers noted how nursing staff could play an active role in a patient’s video visit, for example, by gathering information, fostering connection, and collecting vitals:

By having the LVN meet them first in the (virtual) room, talk about any vitals that they’ve taken at home, or their meds just like in the clinic, and then when she’s got all the information she needs, having the provider come in. I join the room and I hear my LVN, and the Veterans are laughing and talking, and talking about that social kind of things that you would normally do in a face-to-face visit. And my Veterans seem to be very happy with this format.

P3

### Theme 4: Implementing Web-Based Care Requires Institutional and Organizational Support

While providers and other clinical team members play an important role in ensuring the success of video visits, participants also recounted how critical organizational initiatives and support are to implementation and sustainment. Providers noted that when leadership celebrated the success of video visits, it encouraged providers to invest in training and use. They also maintained that clear organization-wide policies and guidelines regarding the use of video visits to provide high-quality care can support growth.

Providers also identified opportunities to enhance video visits by reimagining how health care visits can better incorporate technology. Patient-generated data may inform health care decisions; for example, by including data from a patient’s remote monitoring device, a provider may learn more about a patient’s overall health and well-being.

They also described the opportunity to involve multiple providers in a visit to coordinate and guide clinical care, as this palliative care provider explains:

[There is] more opportunity for combined visits between different specialties, so ways to coordinate visits. For example, to have joint visits with oncology and palliative care is something we do quite frequently in person… And so that something that’s kind of lost virtually.

P28

The previous example underscores the need for institutional support for the technical infrastructure that supports these types of combined visits.

Finally, providers emphasized that for video visits to sustain momentum, organizations and leadership must continue to actively promote the value of video visits for their patients and ensure that video technology is accessible and user friendly. One provider summarized this point of view, stating,

That's why virtual care or telemedicine is so important…. It shouldn't be the Veteran's responsibility to have to struggle to get to the VA. We should make [the VA system] as user friendly as possible, and this is 1 of the mechanisms that allows us to do that.

P10

### Mapping to the ERIC Implementation Science Framework

ERIC implementation strategies are well-known, evidence-based practices that help facilitate the implementation and uptake of interventions. Within each of the 4 identified themes, the interview data highlighted several challenges and recommendations for improving video visit implementation. To further enhance the applicability of our findings, we mapped the challenges and recommendations embedded within each of the identified themes and related strategies to a variety of individual, clinic, and system-level ERIC implementation strategies (Table 2). Our findings suggest that there are several strategies across the individual, clinic, and system levels that may support video visit implementation. At the individual level, strategies can focus on educational materials and meetings that support providers in successfully delivering video visits. Clinic-level strategies are mapped to several areas and should support collaborative engagement, including working with champions and developing a learning collaborative that facilitates education and training needs. Specific strategies at the clinic level include organizing implementation team meetings, examining and refining the workflow, revising professional roles, and having regular consensus discussions. Opportunities to support sustained implementation at the system level should focus on specific strategies, including the involvement of executive boards and developing an implementation blueprint to guide rollout. Quality monitoring tools can be developed to support adherence while still promoting adaptability, clinical innovation, and incentive structures to support adoption.

**Table 2:** Individual-, clinic-, and system-level challenges and strategies that influence providers' adoption of web-based care, mapped to ERIC^a^ strategies [37].

Level^b^	Challenges (from interviews)	Provider recommendations (from interviews)	Corresponding ERIC strategy^b^
Individual	Providers lack experience providing clinical care via video. Certain physical examination maneuvers need to be adapted to an internet-based modality Technical issues can interfere with the flow of a video visit	Develop communication skills to support connections with patients Develop video visit-specific history-taking skills to ensure visit goals are met. Develop new skill sets, like web-based physical examinations Develop skills to troubleshoot technology	Develop educational materials Conduct educational meetings
Clinic	Shortage of education and training on how to implement video visits Conventional team roles do not translate to the video visit workflow Clinic not set up to integrate video visits into schedule Providers do not have time to navigate technical issues in addition to clinic visits	Implement learning sessions and sharing experiences among staff members Clarify staff roles for video visit technical support with patients (test calls, device set up) Use team-based training to support the visit Support team-based care at all levels of the visit, from scheduling (need for video visit, test calls), the digital room (medication reconciliation, tests, vitals), and post-visit follow-up scheduling	Identify and prepare champions Create a learning collaborative Organize clinical implementation team meetings Assess and redesign workflow Revise professional roles Conduct local consensus discussions
System	Organizational infrastructure not equipped to support video visits Some patients need help using technology Lack of incentive to promote video visits Lack of guidance on how to adapt and use technology Need for technical support (patients and providers)	Technology infrastructure improvements to improve care coordination (eg, joint visits across specialties) and unique opportunities (eg, involving family members) Support patients by providing technology Maximize workload credit to support video visits Partner with providers to develop guidance on adopting and integrating technology and data into health care processes Develop scope of practice for staff roles and invest in staff roles to support technical visit needs (for patients and providers)	Purposefully reexamine the implementation Promote adaptability Fund and contract for clinical innovation Alter incentive/allowance structure Involve executive boards Develop and implement tools for quality monitoring Develop an implementation blueprint

^a^ERIC: Expert Recommendations for Implementing Change.

^b^Level of intervention and ERIC strategy mapped by research team based on challenges and recommendations identified in the interview data.

## Discussion

### Principal Findings

This work sought to identify strategies that support VA providers in using video visits effectively. Our findings suggest that the success of video visits relies on the complex coordination of the provider, care team, and organizational factors. As the VA shifts out of the early adoption phase of telehealth and into the long-term maintenance phase, there are new opportunities to support this evolving web-based care landscape. For example, video visits have primarily been used to replicate in-person visits, but there are opportunities to optimize visits for both patients and providers for long-term sustainment. More specifically, not all actions comprising a typical in-person visit must take place synchronously while a physician is conducting a video visit; for example, a patient’s chief complaints, vitals, and screening information can be collected in advance. Hence, engaging patients and clinical staff with new digital modalities to collect and share data has the potential to enhance video visits and should be an organizational priority.

Partnerships with medical organizations and societies in the United States and abroad to develop and refine evidence-based guidelines for optimizing the video visit experience are important mechanisms for moving forward with these strategies. While a few medical associations have released clinical guidelines for telehealth practices, there is still a need to support evidence in the field on optimizing video visit care [41-43].

Our work connecting provider-identified challenges and opportunities with video visits to ERIC implementation strategies yields several helpful insights. First, current training modules may need to be further refined to support education needs at the individual and clinical levels. Our findings also highlight the needs at the organizational level to reexamine the implementation of video visits, including using an implementation blueprint to support health care facilities, clinics, and providers in successful adoption. By promoting adaptability, the local needs of clinics, providers, and patients can be addressed, and funding for innovation encourages stakeholders to take an active role in improving video visit implementation. Organizations will need to potentially redesign the video visit clinic workflow, including revising staff roles, to support sustainment. Informed by the framework in Table 2, organizations may identify strategies to test based on complexity, intervention level (provider versus health care system), and available resources. While strategies for the health care system may lead to sustained change, testing at the individual and clinic level may be more quickly amenable to testing and implementing novel strategies to support providers.

Building long-term infrastructure for technology capacity while preserving patient privacy and security remains a top priority to support video visits. The lack of equitable broadband connectivity in many regions of the United States remains an important technical issue for video visit implementation [44]. Continued investments in technology resources are key to supporting video visits, as the technology is critical to a successful visit. To support staff, the VA has established resource centers to provide training and guidance on how to incorporate web-based care tools into their practice [14,45]. The pandemic led to several regulatory and policy changes that, if maintained post pandemic, may support continued investment in video visits (eg, billing for video visits [46] and state restrictions on care [47]).

There are several limitations to this work. First, our study focused on providers with previous experience using video-based technologies. This decision may impart some selection bias in our findings, as themes may have differed had we included those with less experience in video-based care. Providers with limited experience using video care likely confront a different set of barriers and challenges; understanding their perspectives and experiences with video care is needed to create a fuller account of both the challenges and facilitators of successful video visits.

Second, we only interviewed physicians and nurse practitioners and did not obtain feedback from other clinical team members (eg, nursing staff and schedulers), whose buy-in is necessary for role expansion associated with video-based care promotion. A teamwork approach to web-based care, where technical challenges and troubleshooting with patients are performed by nonclinician members of the care team, could be studied to facilitate video-based care uptake. While this study only focused on provider-based strategies to increase the use of video-based care, we recognize that incorporating and building on patient-focused engagement strategies are also necessary. Though we focused on provider experiences with video care, patients and their engagement with web-based technologies clearly play a role in the “success” of video visits. Furthermore, our interviews are unable to assess the impact of web-based care modalities on patient outcomes. Third, we conducted our interviews during an early period of the COVID-19 pandemic, when many providers were still becoming accustomed to using web-based care. Finally, this study was conducted in the VA, a highly integrated health care system that had an established web-based care infrastructure prior to the pandemic. Providers employed in other health care systems may confront a different set of challenges and barriers, and therefore strategies that work in this setting may have less applicability in others.

### Conclusions

This work summarizes the strategies that providers adopted and identified as important to successfully implement video visits in the VA. Future work should examine the concurrence of the provider experience with executive, program office, and patient perspectives. Additionally, further testing and trialability of these suggested strategies, along with evaluation of their impact on video visit usage and provider satisfaction, would enable the refinement of implementation strategies. Additional research that examines health outcomes associated with web-based care would further support the uptake of these modalities. Ultimately, this would increase their ability to support and facilitate the large-scale implementation and sustainment of video-based care at the VA and other large health care organizations.

## Appendix

Multimedia Appendix 1 Provider Interview Guide.

## Abbreviations

ERIC: Expert Recommendations for Implementing Change
VA: Veterans Affairs
