# Supplementary material for: Opportunities to Enhance the Implementation of Veterans Affairs Video-Based Care: Qualitative Perspectives of Providers from Diverse Specialties
Source: J Med Internet Res. 2023 Apr 24;25:e43314. doi: 10.2196/43314 (PMC10167580; doi:10.2196/43314)
Supplement: Multimedia Appendix 1 [file jmir_v25i1e43314_app1.docx]

**Appendix: Provider Interview Guide**

1. Background: Please tell me about your current role in the VA. How long have you been offering virtual care, either at the VA or elsewhere?
2. As a provider, you may offer both in-person and virtual care to patients. How do you decide when to provide care in person, and when to provide care virtually?
   1. Do you think virtual care is more appropriate for some patients, and not others? If so, what characteristics do these patients share, and why do you think virtual care is (or isn’t) more appropriate for them?
   2. Are some components of care easier to deliver either in person or virtually? If so, which ones and why?
   3. Would you feel comfortable delivering all care virtually? Why or why not?
   4. Are there ever instances where a patient wants virtual care, but you insist that the care be provided in-person? If so, please describe these instances and how you arrive at that decision.
   5. Are there specific guidelines that you use to decide when and how to provide virtual care for patients? If so, please describe.
   6. How much do patient preferences factor into your decision to use virtual care and why?
3. Did you need to learn or develop new skills when you began offering virtual care to your patients? What were these skills, and how did you learn them (or not)?
4. Which resources were or would have been helpful as you were getting started offering virtual care? Which resources would be useful to increase your use of virtual care?
5. Do you find virtual visits with patients to be different from your in-person visits with patients, and if so, please describe why or why not? (probe: communication, establishing rapport and trust). (Only ask if hasn’t been answered through Q 2)
6. Would you like to increase your use of virtual care? Why or why not? If so, are there any organizational factors that make you more or less likely to increase your use of virtual care? Are there ways you might be encouraged to interact with patients virtually more often?
7. Do you face barriers to offering virtual care more often? If so, what are these barriers?
8. Due to the spread of COVID-19, the VA has ramped up its use of telehealth. Please describe your experience with this effort, and how it impacted your use of telehealth and possibly changed some of your impressions of telehealth.
9. VA recently implemented a digital divide consult. Are you aware of this initiative? How do you think it might change your use of virtual care, if at all?
10. We are interested in understanding whether or not patients with disabilities, including hearing, vision, and mobility, experience barriers to receiving virtual care. As a provider who uses virtual care, have you observed any barriers to greater usage of telehealth modalities among these groups? Do you have any other observations on this topic that you’d like to share?

**VA Tablet Program-specific Question(s)**

1. Do you currently, or have you in the past issued tablets to patients? Do you find them to be beneficial for patient care? Why or why not?
   1. Have you noticed a difference in virtual visits with patients who have their own devices versus those who use VA-issued tablets? (probe: technology challenges, patient’s comfort with technology)
   2. How do you identify whether a patient might be a good candidate for a tablet?
   3. What have you learned about tablet users/non-users
